# Comparative metabolic ecology of tropical herbivorous echinoids on a coral reef

**DOI:** 10.1371/journal.pone.0190470

**Published:** 2018-01-18

**Authors:** Levi S. Lewis, Jennifer E. Smith, Yoan Eynaud

**Affiliations:** Center for Marine Biodiversity and Conservation, Scripps Institution of Oceanography, University of California San Diego, San Diego, California, United States of America; Universidade Federal do Rio de Janeiro, BRAZIL

## Abstract

**Background:**

The metabolic rate of consumers is a key driver of ecosystem dynamics. On coral reefs, herbivorous echinoids consume fleshy algae, facilitating the growth of reef-building calcified organisms; however, little is known about differences among species in their metabolic and functional ecology. Here, we used log-linear (log-log) regression models to examine the allometric scaling of mass and routine metabolic rate for five common herbivorous echinoids on a Hawaiian coral reef: *Echinothrix calamaris*, *E*. *diadema*, *Echinometra matthaei*, *Heterocentrotus mammillatus*, and *Tripneustes gratilla*. Scaling relationships were then contrasted with empirical observations of echinoid ecology and general metabolic theory to broaden our understanding of diversity in the metabolic and functional ecology of tropical herbivorous echinoids.

**Results:**

Test diameter and species explained 98% of the variation in mass, and mass and species explained 92.4% and 87.5% of the variation in individual (*I*) and mass-specific (*B*) metabolic rates, respectively. Scaling exponents did not differ for mass or metabolism; however, normalizing constants differed significantly among species. Mass varied as the cube of test diameter (*b* = 2.9), with HM exhibiting a significantly higher normalizing constant than other species, likely due to its heavily-calcified spines and skeleton. Individual metabolic rate varied approximately as the 2/5 power of mass (*γ* = 0.44); significantly smaller than the 3/4 universal scaling coefficient, but inclusive of 2/3 scaling. *E*. *calamaris* and *H*. *mammillatus* exhibited the lowest normalizing constants, corresponding with their slow-moving, cryptic, rock-boring life-history. In contrast, *E*. *calamaris*, *E*. *diadema*, and *T*. *gratilla*, exhibited higher metabolic rates, likely reflecting their higher levels of activity and ability to freely browse for preferred algae due to chemical anti-predator defenses. Thus, differences in metabolic scaling appeared to correspond with differences in phylogeny, behavior, and ecological function. Such comparative metabolic assessments are central to informing theory, ecological models, and the effective management of ecosystems.

## Introduction

The metabolic rates of organisms drive numerous ecological dynamics. Metabolic rates can inform us about biomass production, ontogenetic growth, mortality, interspecific interactions (i.e., predation and competition), species diversity, energy fluxes, and population and trophic dynamics [1, 2]. Relative rates of production and consumption among trophic levels in an ecosystem determine biomass accumulation, community structure, habitat complexity, and ecological function [2]. Consumers therefore can exert strong ecological effects: predation by asteroids and herbivory by echinoids are often dominant, structuring forces in benthic marine ecosystems, both intertidal and subtidal, and in tropical temperate, and polar seas [3–7]. Due to their strong interaction strengths, it is important that we understand how the ecological functions of echinoid species and communities vary in nature to better inform conservation and management efforts in associated ecosystems [8].

Coral reefs, in particular, are sensitive to production-consumption dynamics: they develop and persist under prolonged stable conditions where the consumption-production ratios for fleshy algae remain high, thus limiting algal proliferation and facilitating the dominance of calcifying organisms (e.g., scleractinian corals and coralline algae) and net reef accretion [9]. Enhanced production (e.g., via nutrient pollution) or reduced consumption (e.g., via overfishing of herbivores) of fleshy algae on coral reefs disrupts this crucial balance, leading to wide-spread loss of live coral cover and the degradation of coral reef ecosystems [10, 11]. The transition to a fleshy-algal dominated system disrupts many of the key process that generate the characteristic high complexity and diversity of coral reefs; thus reefs erode into low-complexity systems with greatly diminished biodiversity and productivity [12]. The most important grazers in coral reef ecosystems are fishes and echinoids [13], and the loss of these herbivores (due to fishing and disease) has led to numerous wide-spread declines in live coral cover, both globally and throughout Hawaii, that have been associated with the expansion of fleshy algae [10, 14–18]. As a result, herbivore protections [19], and even enhancement of echinoid populations via aquaculture [20, 21], have become important strategies for coral reef conservation and restoration, especially in Hawaii.

The net function of an herbivore community is influenced by its structure, biomass and metabolism, and the metabolism of individual community members is most strongly influenced by body size (mass) and temperature as described by several metabolic theories [1]. Though much focus has been placed on the many unique roles that different herbivorous fishes may play in coral ecosystems [22, 23], less is known about taxon-specific variation in ecological traits of diverse communities of echinoids, such as those found in the tropical Pacific and Indian Oceans [24, 25]. In particular, little is known about how different echinoid species utilize resources, or how different species fit into metabolism-based models of coral reef ecosystem dynamics [2, 26]. Such information is important for understanding variation in ecological functions and predicting ecosystem responses of coral reefs to local and global stressors [27, 28].

Several echinoid species exist in sympatry on Hawaii’s shallow coral reefs, with unique communities being defined by the relative abundances of 4 common genera: *Heterocentrotus*, *Echinothrix*, *Tripneustes and Echinometra* [24, 29]. Echinoids within these genera exhibit unique morphologies (e.g., tests and spines) and behaviors (e.g., burrowing, movement, predator avoidance, and feeding). For example, grazing behaviors inferred from gut contents and algal surveys have revealed differences in diets that could be indicative of selectivity [24]. For example, *Echinothrix sp*. appear to prefer to feed on simple, fleshy algal forms [30], whereas *Tripneustes sp*. may exhibit a preference for leathery, chemically-defended brown algae that are avoided by diadematids [31]. *Echinothrix sp*. and *Tripneustes sp*. express venomous spines and pedicellariae that allow them to avoid predation while feeding in the open [32, 33]. In contrast, *Echinometra sp*. and *Heterocentrotus sp*. do not appear to exhibit effective chemical defenses, and thus must hide in burrows or shallow water to avoid predation [24, 34, 35]. By exploring taxon-specific variation in the mass-scaling of metabolism among several echinoid species, we can provide a better understanding of how species might differ functionally and how metabolism relates to phylogenetic history or might co-evolve with variation in morphology and life-history strategies.

Here, we used standard log-linear (log-log) regression models to describe variation in the allometric scaling of mass and metabolism for five dominant echinoid species in the Hawaiian Islands. If echinoids exhibit diversity in mass and metabolism, we predicted that allometric scaling parameters would differ significantly among species. If differences were determined by phylogenetic history, we predicted that more genetically similar taxa (e.g., within a genus or family) would express more similar scaling relationships, whereas significant within-group variation would suggest functional divergence [36]. Furthermore, if echinoid measurements were precise, we expected mass (∝ volume) to scale as the cube (*b* = 3.0) of test diameter [37]; and if metabolic scaling of echinoids matches global mean values across most animal phyla, we predicted mass-scaling of echinoid metabolism to match the ‘universal’ scaling exponent (*γ* = 0.75, Kleiber’s Law) of the Metabolic Theory of Ecology (MTE) [38]. By describing and contrasting test-mass and mass-metabolism relationships among several coexisting echinoid species, we aimed to shed light on metabolic and ecological diversity within this important guild and provide estimates of key metrics necessary for parameterizing metabolic-based community and ecosystem models.

## Methods

This study was conducted in consultation with the Maui Division of Aquatic Resources (R. Sparks and D. White) under DAR permit number SAP2014-42.

### Study site

Field work was conducted on nearshore (2–7 m depth) fringing coral reefs on the island of Maui, Hawaii (20°56’19"N, 156°41’35"W). The cover of live corals in Hawaii has declined rapidly over the last several decades, with many reefs showing signs of recent degradation and algal overgrowth [39, 40], concurrent with historic and on-going fishing activities [14, 15]. Marine herbivores (e.g., fishes and echinoids) are important in Hawaii as algal grazers, especially given the high inputs of nutrient-laden groundwater and runoff [17, 41, 42] that stimulate fleshy algal blooms at the expense of live corals [43, 44]. Fourteen species of echinoids are found in Hawaii, with nearshore communities often dominated by herbivorous taxa within the genera *Heterocentrotus*, *Echinometra*, *Echinothrix*, and *Tripneustes*. Nearshore echinoid communities can reach densities > 70 ind./m^2^ [24] and biomass > 900 g/m^2^ [29], both of which generally decline with depth. Echinoids in this study were collected from within the Kahekili Fishery Management Area (KHFMA) where herbivores, including echinoids, have been protected since 2009 to limit algal blooms and promote coral growth (Hawaii DLNR §13–60.7) [19, 45]. This site was excellent given the abundance and diversity of taxa available; however, given the strict protections for herbivores in the region, all echinoids were assayed and released at the site of collection within 24 hours, and not starved.

### Metabolic assays

**Fig 1:**
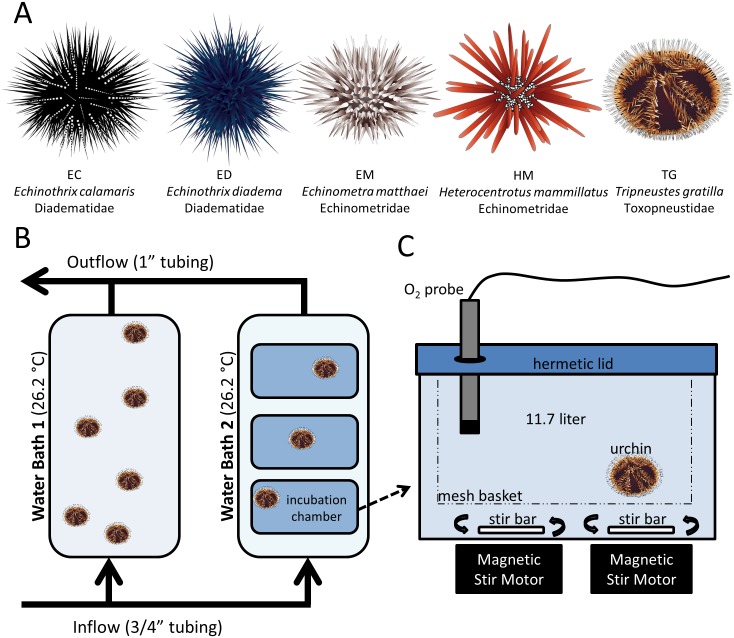
Study design. (A) Herbivorous echinoids used in this study, (B) design of portable flow-through acclimation and assay water baths, (C) design of portable respirometry chamber. Echinoid artwork by Adi Khen.

In June-July 2014, we measured routine oxygen consumption rates to compare differences in metabolic activity among five echinoid species: *Echinothrix calamaris* (EC), *Echinothrix diadema* (ED), *Echinometra matthaei* (EM), *Heterocentrotus mammillatus* (HM), and *Tripneustes gratilla* (TG) (Fig 1a). These species represented three echinoid families common to coral reefs world-wide: Diadematidae (EC, ED), Echinometridae (EM, HM), and Toxopneustidae (TG). Species differed greatly in size with (mean ± SE) test diameters from 4.1 ± 1 (EM) to 7.0 ±0.1 (TG) cm, masses from 32.1± 1.6 (EM) to 154.3 ± 8.3 (HM) g, and test volumes from 17.8 ± 0.67 (EM) to 92.0 ± 4.3 ml (TG) (S1 Fig). Metabolic assays were conducted in static (constantly-stirred) chambers using a custom, portable respirometry lab (Fig 1b and 1c) at the Maui Ocean Center (MOC) located in Ma’alaea, Maui. Echinoids were collected from fringing reefs in West Maui and transported to the MOC in a continually-aerated 142 L cooler (transport time = 30 min.). At the MOC, the cooler was immediately connected to flow-through seawater and echinoids were allowed to acclimate in the dark for 60 min (Fig 1b).

After acclimation, one individual was gently placed in each of three clear plastic incubation chambers containing a plastic mesh basket and 11.7 liters of fresh seawater that was stirred constantly by 2 rotating stir bars (Fig 1b). Metabolic chambers were housed in a 142 liter cooler and bathed in ambient flow-through seawater to maintain a constant temperature of 26.2°C (SD = 0.1). Initial temperature and oxygen measurements were then taken in each chamber, the chambers were hermetically-sealed, and the cooler closed. All temperatures (to 0.1°C) and dissolved oxygen concentrations (to 0.01 mg/l) were measured using a Hq40d Hach portable meter fitted with a luminescent dissolved oxygen optode and temperature probe (Hach Company, USA). Probes were calibrated to 100% air saturation (using air saturated with water vapor, per Hach instructions) once each day prior to the first assay and monitored for consistency.

After 60 min. (based on pilot studies), oxygen and temperature were measured again through a hole in the hermetically-sealed lid (Fig 1c). Echinoids were then removed, maximum test diameters (to 0.1 cm using calipers) and wet mass (to 0.1 g by placing the individual in a seawater-filled container on a tared digital balance) measured, and then placed in a separate holding tank. Volumetric displacement of echinoids (modeled as a hemisphere) was always less than 1% of total chamber volume. Metabolic chambers were then rinsed, refilled with fresh seawater, and two additional 60 min. assays conducted (each with three additional individuals) for a total of nine individuals per species. To account for background (microbial) respiration, six 60-min. control assays were conducted daily (3 each before and after), and oxygen consumption rates of echinoids were corrected by subtracting the corresponding daily mean microbial respiration rate. Mean (± SE) microbial respiration was 0.27 ± 0.03 mg/h (0.02 mg/liter/h) and remained significantly lower than echinoid treatments (S2 Fig). Examination of the three replicate assays (n = 3 individuals per replicate) for each species (i.e., day) indicated minimal differences in measured rates between replicate assays (S3 Fig). Individual metabolic rate (*I*) was calculated by multiplying the change in oxygen concentrations by the volume of the chamber and dividing by the elapsed time (mgO_2_/h). Biomass-specific metabolic rate (*B*) was calculated by dividing *I* by the wet mass of the corresponding individual (mgO_2_/g/h).

Overall, our assays followed general best practices with respect to volume, mixing, measurement, temperature, and controls for microbes; specifically for static chambers with stir bars which are appropriate and commonly used for echinoderms and other benthic invertebrates [46–49]. Echinoids were collected from within a marine protected area (Hawaii DLNR §13–60.7) where they were most abundant; therefore, each species was assayed on a separate day and all individuals returned to the site of collection the following morning. Because organisms were only held for 24 hours in the laboratory (total), we used unstarved individuals and the rates we measured included natural variation in recent feeding behavior (postprandial metabolism). Variation in postprandial metabolism may increase metabolic rates (via specific dynamic action) of organisms in, and recently collected from, the field, though less so for herbivorous echinoids than many other taxa [50]. Though starvation of organisms is commonly used to reduce natural variation, we note that starvation “is itself an experimental condition and not a control, which affects the results” [51]; thus starvation systematically ignores an "integral part of an organism’s energy budget" [50]. Thus the routine metabolic rates reported here may have been influenced by some degree of natural intra- and inter-specific variation in recent feeding behavior, as would be observed in more ecologically-relevant metabolic measurements such as field metabolic rates (FMR) [52].

#### Allometric power functions

Mass and metabolic rates were modeled using the standard power function

$$y\;=\;ax^{b}$$ (1)

where *a* is the normalizing constant and *b* is the scaling exponent. Log-log transformations of this model are linear (log-linear) and well-suited for allometric scaling of mass and metabolism due to their simple solutions for parameter estimates (*b* = slope, *a* = 10^intercept^) [1, 37, 53]:

log(y)=b*log(x)+log(a). (2)

Eq (2) was modified to fit each of the three modeled log-linear relationships below for each echinoid species (*i*):

$$\text{log}\;\left(M_{i}\right)\;=\;b\;*\;log(D_{i})+\;log\;\left(M_{o,i}\right)$$ (3)

where *M*_*i*_ is the wet mass (g) and *D*_*i*_ the test diameter (cm) of echnoid species *i*,

$$\text{log}\;\left(I_{i}\right)\;=\;\gamma \;*\;log(M_{i})+\;log\;\left(I_{o,i}\right)$$ (4)

where *I*_*i*_ is the individual metabolic rate of species *i*, (mgO_2_/h) and

$$\text{log}\;\left(B_{i}\right)\;=\;\alpha \;*\;log{(M}_{i})+\;log\;\left(B_{o,i}\right)$$ (5)

where *B*_*i*_ is the biomass-specific metabolic rate of species *i* (mgO_2_/h/g).

No significant differences among species in the scaling exponents (*b*, *γ*, and α) for any model (*M*_*i*_, *I*_*i*_, nor *B*_*i*_, respectively) was observed (see Results); therefore, log-linear slopes were considered homogeneous among species. Normalizing constants (intercepts), however, differed significantly among species for all models. Expanded parameter estimates were used to compare intercepts among species; thus each species-specific model (*M*_*i*_, *I*_*i*_, *B*_*i*_) included a global intercept (c_1,_ c_2,_ c_3_) and species-specific modifier ($\Delta_{i}^{M},\Delta_{i}^{I},\Delta_{i}^{B}$), respectively, satisfying the equations:

$$M_{o,i}\;=\;10^{\left(\Delta_{i}^{M}+c_{1}\right)}$$ (6)

$$I_{o,i}\;=\;10^{\left(\Delta_{i}^{I}+c_{2}\right)}$$ (7)

$$B_{o,i}\;=\;10^{\left(\Delta_{i}^{B\;}+c_{3}\right)}$$ (8)

#### Statistical analyses

We used general linear models (with heterogeneous slopes), analysis of covariance (ANCOVA, with homogeneous slopes), and linear regression to statistically examine log-linear relationships and scaling parameters (scaling exponent = slope, normalizing constant = 10^intercept^, Eq 2,), and test for differences among echinoid species [53]. Echinoid wet mass (*M*, g) was modeled as a function of test diameter (*D*, cm), and individual (*I*, mgO_2_/h) and biomass-specific (*B*, mgO_2_/g/h) oxygen consumption rates were each modeled as functions of *M*. Echinoid species was included as a fixed effect in each model. To assess the effects of calcified skeleton on scaling relationships, oxygen consumption rate was also modeled as a function of test volume. Normalizing constants were compared among species using 95% confidence intervals. Parametric assumptions were evaluated for each test using Q-Q and residual plots—any departures appeared small and the methods used are robust for balanced designs [54]. Statistics were conducted using JMP Pro 12.01.1 (SAS Institute Inc., Cary, NC, USA) and R 3.2.0 (R Core Team, 2016).

## Results

General linear models of log-log transformed allometric relationships (*M* vs *D*, *I* vs. *M*, and *B* vs. *M*) indicated strong relationships (R^2^ = 0.98, 0.92, 0.89, respectively) and significant (P < 0.05) differences among echinoid species for all allometric relationships (Table 1). Scaling exponents (slope = *b*, γ and α), however, did not differ among species; therefore, slopes were treated as homogeneous across all species and relationships were subsequently modeled using ANCOVA by removing the interaction terms.

**Table 1:** Results of full log-linear (log-log) regression models including interaction terms (testing for heterogeneous slopes).

Model	Source/Factor	DF	SS	MS	F	P	R^2^_adj_
*M*	Model	9	3.512	0.390	234.81	<0.001	0.979
	Error	35	0.058	0.002			
	C. Total	44	3.570				
	Species	4	0.402		60.43	<0.001	
	Log(*D*)	1	0.186		111.65	<0.001	
	Log(*D*)*Species	4	0.002		0.29	**0.884**	
*I*	Model	9	5.313	0.590	57.59	<0.001	0.920
	Error	35	0.359	0.010			
	C. Total	44	5.672				
	Species	4	0.424		10.34	<0.001	
	Log(*M*)	1	0.057		5.57	0.024	
	Log(*M*)*Species	4	0.023		0.56	**0.693**	
*B*	Model	9	3.082	0.342	33.41	<0.001	0.869
	Error	35	0.359	0.010			
	C. Total	44	3.440				
	Species	4	0.424		10.34	<0.001	
	Log(*M*)	1	0.068		6.59	0.015	
	Log(*M*)*Species	4	0.023		0.56	**0.693**	

Models are echinoid mass (*M*, g) vs. test diameter (*D*, cm), individual metabolic rate (*I*, mgO_2_/h) versus *M*, and biomass-specific metabolic rate (*B*, mgO_2_/g/h) vs *M*. P-values of interaction terms (bold) were high and non-significant, thus final models were run using ANCOVA assuming homogeneous slopes.

### Mass versus test-diameter

**Fig 2:**
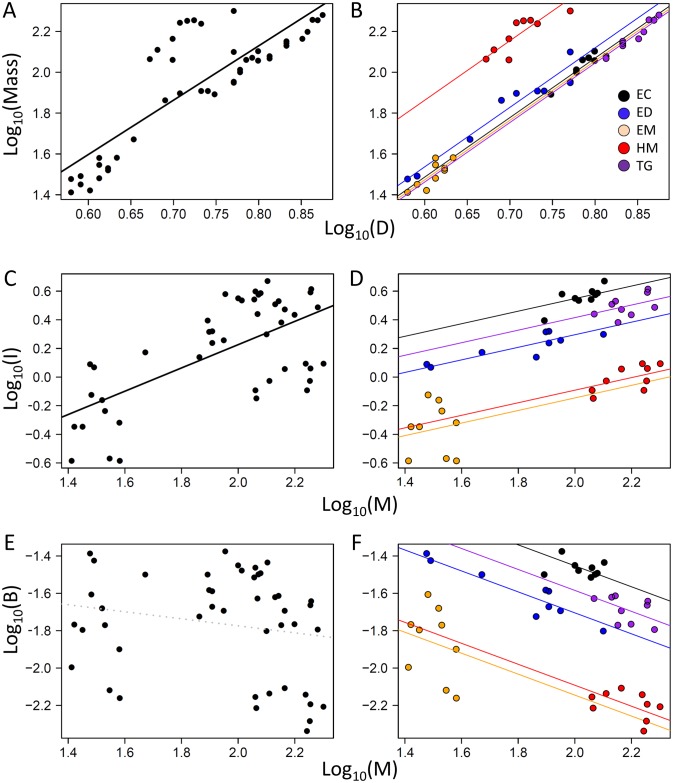
Allometric relationships of echinoid mass and metabolism. Mass (*M*, g) vs. test diameter (*D*, cm) (a-b); and individual (*I*, mgO_2_/h) (c-d) and biomass-specific (*B*, mgO_2_/g/h) (e-f) metabolic rates vs. mass. Figures on the left (a,c,e) include all individuals pooled; figures on the right (b,d,f) include species as fixed factors (slopes = homogenous). All data were Log_10_(x) transformed and lines represent ordinary least-square linear fits of log-log transformations (Table 2). Echinoid species codes as in Fig 1a.

**Fig 3:**
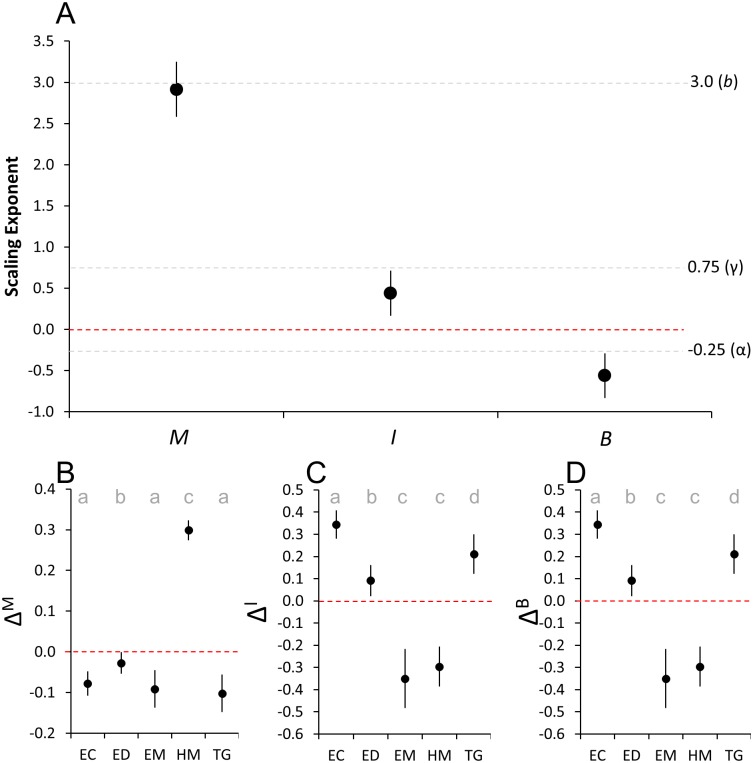
Allometric scaling parameters for mass and metabolism. Plots of (a) estimated mass-scaling exponents and predicted values (grey-dashed lines) for mass (*b* = 3.0), individual metabolic rate (γ = 0.75) and mass-specific metabolic rate (*α* = -0.25), and (b-d) species-specific normalizing coefficient modifiers ($\Delta_{i}^{M},\Delta_{i}^{I},\Delta_{i}^{B}$). Letters in (b-d) indicate groupings based on 95% confidence intervals. All error bars = ±95% confidence intervals. Red-dashed lines = 0. Echinoid species codes as in Fig 1a.

Test diameter explained 69.3% (alone, linear regression) and 98.1% (including species as a fixed factor, ANCOVA) of the variation in echinoid mass (Table 2, Fig 2a and 2b). Echinoid mass scaled as the approximate cube (*b* = 2.91 ± 0.17 SE) of test diameter for all species (Table 2, Fig 3a). Though scaling exponents (slopes) did not differ among species, differences in normalizing constants (*a* = intercepts) were highly significant (Table 2). Differences in intercepts for EC, EM, and TG were small and non-significant; however, ED was slightly heavier and HM much heavier, with significantly greater intercepts than all other species (Table 2, Fig 3b).

**Table 2:** Results of log-linear regression (ignoring species) and analysis of covariance (ANCOVA, homogeneous slopes) models using log-log transformations. Echinoid species codes as in Fig 1a.

Metric	Factors	N	K	F	P	R^2^adj	Parameter	Value	SE	t	P	95% L	95% U	
*M*	Log(*D*)	45	5	100.33	<0.0001	0.693	Intercept	0.000	0.195	-0.002	0.998	-0.394	0.393	
							slope	**2.662**	0.266	10.020	**<0.001**	2.126	3.199	***
	Log(*D*)+Sp.	45	5	455.77	<0.0001	0.981	Intercept(*c*_*1*_)	-0.184	0.121	-1.530	0.135	-0.428	0.060	
							slope(*b*)	**2.914**	0.165	17.650	**<0.001**	2.580	3.248	***
							EC	-0.078	0.015	-5.230	<0.001	-0.108	-0.048	a
							ED	-0.028	0.013	-2.100	0.042	-0.054	-0.001	b
							EM	-0.091	0.023	-3.980	<0.001	-0.138	-0.045	a
							HM	0.299	0.012	24.750	<0.001	0.274	0.323	a
							TG	-0.102	0.023	-4.490	<0.001	-0.148	-0.056	c
*I*	Log(*M*)	45	5	30.57	<0.0001	0.402	Intercept	-1.399	0.288	-4.850	<0.001	-1.980	-0.817	
							slope	**0.813**	0.147	5.530	**<0.001**	0.516	1.109	***
	Log(*M*)+Sp.	45	5	108.09	<0.0001	0.924	Intercept (*c*_*2*_)	-0.672	0.262	-2.570	0.014	-1.202	-0.143	
							slope(γ)	**0.438**	0.135	3.250	**0.002**	0.166	0.711	**
							EC	0.344	0.032	10.880	<0.001	0.280	0.408	a
							ED	0.091	0.035	2.640	0.012	0.021	0.161	b
							EM	-0.350	0.066	-5.300	<0.001	-0.484	-0.217	c
							HM	-0.296	0.044	-6.670	<0.001	-0.386	-0.206	c
							TG	0.211	0.044	4.810	<0.001	0.122	0.300	d
*B*	Log(*M*)	45	5	1.63	0.209	0.014	Intercept	-1.399	0.288	-4.850	<0.001	-1.980	-0.817	
							slope	**-0.187**	0.147	-1.280	**0.209**	-0.484	0.109	NS
	Log(*M*)+Sp.	45	5	62.49	<0.0001	0.875	Intercept (*c*_*3*_)	-0.672	0.262	-2.570	0.014	-1.202	-0.143	
							slope(*α*)	**-0.562**	0.135	-4.170	**<0.001**	-0.834	-0.289	***
							EC	0.344	0.032	10.880	<0.001	0.280	0.408	a
							ED	0.091	0.035	2.640	0.012	0.021	0.161	b
							EM	-0.350	0.066	-5.300	<0.001	-0.484	-0.217	c
							HM	-0.296	0.044	-6.670	<0.001	-0.386	-0.206	c
							TG	0.211	0.044	4.810	<0.001	0.122	0.300	d

Metrics: *M* = mass (g), *I* = individual metabolic rate (mgO_2_/h), *B* = biomass-specific metabolic rate (mgO_2_/h/g). Factors: *D* = test diameter (cm), *M* = mass (g), Urchin species = EC, ED, EM, HM, TG (Fig 1a). For ANCOVAs (sp. = factor), global slopes (*b*, *γ*, *α*) and intercepts (c_1_, c_2_, c_3_) are provided, as well as the associated species-specific intercept modifiers ($\Delta_{i}^{a},\Delta_{i}^{I},\Delta_{i}^{B}$) represented by species codes.

Asterisks reflect the significance of scaling exponents (slopes) and letters denote differences among species in normalizing constants (intercepts). Echinoid species codes as in Fig 1a.

### Metabolism versus mass

Echinoid mass alone explained 40.2% (regression), and 92.4% (ANCOVA) with species as a fixed factor, of the variation in echinoid individual metabolic rate (*I*) (Table 2, Fig 2c and 2d). Individual metabolic rate scaled as the approximate 2/5 exponent (*γ* = 0.44 ± 0.14 SE) of mass for all species (Table 2, Fig 3a), differing significantly from 3/4 (0.75) predicted by Kleiber’s Law and MTE [38]. Differences among species’ intercepts (i.e., normalizing constants) were highly significant with EC>TG>ED>HM = EM (Table 1, Fig 3b). Intercepts for EC, TG, and ED (${mean\;\Delta }_{i}^{I}\;=\;0.22$) were all much higher than those of the echinometrids HM and EM (${mean\;\Delta }_{i}^{I}\;=\;-0.32$) (Fig 3b).

Echinoid mass alone explained 0.014% (regression), and 87.5% (ANCOVA) with species as a fixed factor, of the variation in echinoid biomass-specific metabolic rate (*B*) (Table 1, Fig 2e and 2f). Echinoid mass-specific metabolic rates scaled as the approximate -1/2 exponent (α = -0.56 ± 0.14 SE) of mass for all species (Table 2), also differing significantly from the -1/4 (-0.25) predicted by MTE [1]. Given that *B* is directly related to *I* (e.g., *B* = *I/M*), differences among species in intercepts (normalizing constants) were equivalent and highly significant (Table 2, Fig 3d).

Allometric scaling of individual metabolism (*I*) versus test volume (*V*, cm^3^) yielded results similar to those for echinoid mass (Table 3, S4 Fig). The volume-based scaling exponent (γ = 0.49 ± 0.14 SE) remained low and similar to the mass-based value (γ = 0.44) (Table 3). Unlike mass, the upper 95% confidence limit (γ = 0.77) overlapped slightly with Kleiber’s value (0.75). Intercepts differed significantly among species and appeared similar to differences observed for mass-based models, except that HM exhibited a significantly higher intercept ($\Delta_{i}^{I}\;=\;-0.16+/-\;0.03$) than EM ($\Delta_{i}^{I}\;=\;-0.37+/-\;0.06$), indicating a faster metabolic rate of HM’s soft tissues previously masked by its robust, metabolically-inactive skeleton (Table 3, S4 Fig).

**Table 3:** Results of analysis of covariance (ANCOVA, homogeneous slopes) model on log-log transformed individual metabolic rate (*I)* and volume (*V*).

Metric	Predictor	N	K	F	P	R^2^adj	Parameter	Value	SE	t	P	95% L	95% U	
I	Log(V)+Sp.	45	5	114.50	<.0001*	0.928	Intercept (c_2_)	-0.762	0.258	-2.950	0.005	-1.284	-0.240	
							slope(γ)	**0.493**	0.135	3.650	**<0.001**	0.220	0.766	***
							EC	0.298	0.036	8.170	<0.001	0.225	0.372	a
							ED	0.087	0.032	2.680	0.011	0.021	0.152	b
							EM	-0.366	0.056	-6.510	<0.001	-0.480	-0.252	c
							HM	-0.162	0.030	-5.460	<0.001	-0.222	-0.102	d
							TG	0.143	0.056	2.560	0.015	0.030	0.256	b

Metrics: *I* = individual metabolic rate (mgO_2_/h), *V* = test volume (cm^3^). Global scaling exponent (*γ*) and intercept (*c*) are provided along with the associated species-specific intercept modifiers ($\Delta_{i}^{I}$) represented by species codes (EC, ED, EM, HM, TG; Fig 1a).

Asterisks reflect the significance of scaling exponents (slopes) and letters denote differences among species in normalizing constants (intercepts).

## Discussion

**Fig 4:**
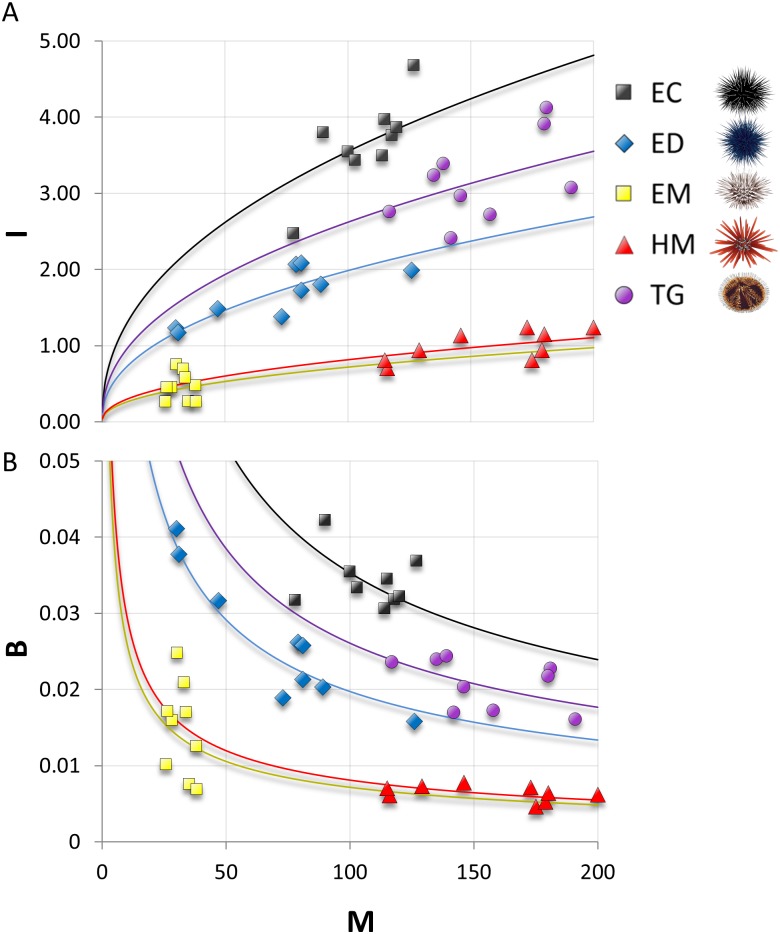
Back-transformed (raw) metabolic scaling relationships. Echinoid mass (*M* in g) versus (A) individual metabolic rate (*I* in mgO_2_/h), and (B) biomass-specific metabolic rate (*B* in mgO_2_/g/h). Lines represent corresponding scaling functions from Table 4. Urchin codes as in Fig 1a.

Here, we described variation in the allometric scaling of mass and metabolism for five species of tropical herbivorous echinoids common to coral reefs in Hawaii and around the globe. This trophic guild plays an important role in coral reef ecosystems by consuming fleshy algae and facilitating the abundance and growth of reef-building corals and coralline algae [16, 55, 56]. Differences among species were both statistically and ecologically significant. HM exhibited a unique morphology, and EC, ED, and TG exhibited the highest metabolic rates (both individual and mass-specific) while both echinometrids (EM and HM) exhibiting the lowest. Such metabolic contrasts can greatly advance our understanding of the diversity of ecological functions within and among trophic guilds and communities. For example, metabolic rates can inform us about biomass production, ontogenetic growth, mortality, interspecific interactions, species diversity, energy fluxes, and population and trophic dynamics [1, 2]. The development and testing of metabolic theory is dependent on individual studies, like ours, that contrast metabolic rates across a variety of taxa [57, 58]. By describing the comparative metabolic ecology of several important tropical herbivorous echinoids, we advance our understanding of the metabolic and trophic ecology of this important herbivore guild (Table 4, Fig 4).

**Table 4:** Summary of species-specific log-linear and back-transformed allometric scaling models for tropical echinoids. Echinoid species codes as in Fig 1a.

Metric(y)	Spp	Factor	log(y) = b*log(x) + log(a)	y = a*x^b^
*M*	EC	*D*	2.915*log(*D*) − 0.262	0.548*D^2.915^
	ED	*D*	2.915*log(*D*) − 0.212	0.614*D^2.915^
	EM	*D*	2.915*log(*D*) − 0.276	0.531*D^2.915^
	HM	*D*	2.915*log(*D*) + 0.115	1.302*D^2.915^
	TG	*D*	2.915*log(*D*) − 0.287	0.518*D^2.915^
*I*	EC	*M*	0.439*log(*M*) − 0.329	0.263*M^0.439^
	ED	*M*	0.439*log(*M*) − 0.581	0.263*M^0.439^
	EM	*M*	0.439*log(*M*) − 1.023	0.095*M^0.439^
	HM	*M*	0.439*log(*M*) − 0.969	0.108*M^0.439^
	TG	*M*	0.439*log(*M*) − 0.461	0.347*M^0.439^
*B*	EC	*M*	-0.562*log(*M*) − 0.329	0.470*M^-0.562^
	ED	*M*	-0.562*log(*M*) − 0.581	0.263*M^-0.562^
	EM	*M*	-0.562*log(*M*) − 1.023	0.095*M^-0.562^
	HM	*M*	-0.562*log(*M*) − 0.969	0.108*M^-0.562^
	TG	*M*	-0.562*log(*M*) − 0.461	0.347*M^-0.562^

### Metabolic ecology of Hawaiian echinoids

The taxonomic variation in echinoid metabolism we have described is supported by several observations of the unique life history strategies and feeding behaviors of these species. EC exhibited the highest metabolic rates of all echinoids assayed. This species is known to be a voracious consumer of algae [30] and is regularly observed feeding in the open, both day and night [24]. Furthermore this species is highly active, exhibiting rapid transit across the reef and rapid waving of its spines upon disturbance. The venomous spines likely deter predators, thus allowing EC to freely search of preferred algal prey. For example, members of the diadematid family, in general, appear to prefer diets of simple fleshy algae (e.g., *Codium*, *Padina*, *Hydrolcathrus*, and filaments) and avoid leathery, chemically-defended brown algae (e.g., *Sargassum* and *Turbinaria)* [30]. Though the congener, ED, also exhibited relatively high metabolic rates, these were significantly lower than EC. This difference in metabolism between these species matched observations of their distinct behaviors in the field. For example, ED was observed more often in burrows than EC and, though it exhibited some motion upon disturbance, it was clearly slower and less active than EC. Thus, these two diadematids exhibited relatively high metabolic rates that varied with life history and corresponded with high consumption rates, chemical defenses, and a preference for more palatable algae.

Like the diadematids, TG (a toxopneustid) exhibited a relatively high metabolic rate and was observed feeding in the open both day and night [32, 59]. Furthermore it is known to be a voracious consumer of algae, so effective that it is used as a biological (algae) control agent [20, 21]. Unlike diadematids, however, this species lacks long venomous spines and exhibits slower movements. Instead of long spines, TG exhibits short venomous globiferous pedicellariae that deter predators and allow it to, like EC, feed in the open both day and night, thus it is able to freely seek out preferred algae [32, 33]. Unlike the diadematids, TG readily consumes chemically-defended brown algae that are avoided by diadematids [31]. Though phenolic compounds in these brown algae are believed to deter grazers [60], TG may preferentially consume (and thrive) on algae with higher phenolic concentrations [31]. Thus TG exhibited a high metabolic rate that corresponded with a high grazing rate, chemical defenses, and a preference for chemically-defended algae.

In contrast to the diadematids and TG, the echinometrids, EM and HM, exhibited much lower metabolic rates. Previous studies indicate that these low metabolic rates correspond with EM’s generalist diet and behavior [34, 61]. Furthermore, both echinometrid species are known to chew on and erode carbonate reefs by feeding on endolithic and crustose coralline algae and excavating burrows in which they hide during the day. Movements are limited, with individuals grazing on drift algae or benthic algae on burrow edges at night [24, 34, 35]. Thus HM and EM exhibited low metabolic rates that corresponded with a burrowing lifestyle, consumption of low quality foods, and a lack of effective predator defenses. In sum, the taxon-specific differences in metabolism we observed corresponded with differences in the ecological strategies and feeding behaviors exhibited by each species.

### Echinoid mass

Test diameter alone explained the majority (70%) of variation in echinoid mass; however including species identity increased explanatory power to > 98%. The allometric scaling exponent (*b* = 2.914 ± 0.165 SE) for the estimation of mass from diameter did not differ among urchin species, and was similar to our prediction (*b* = 3) given that volume (∝ mass) is a cubic function of linear measurements (e.g., diameter)[37]. Four of the five echinoid species exhibited similar normalization constants (*M*_*o*_) and scaling functions, with HM being the one exception. Though HM is in the same family as EM, its large-spined morphology is similar to distantly-related "rough-spined" pencil echinoids of the family Cidaridae. It appears that HM’s unique, heavily-calcified spines greatly increased its normalization constant (i.e., relative mass) and that echinoid morphology is more important for accurate mass-scaling than phylogenetic history, suggesting that significant morphological specialization has occurred independent of phylogeny.

The mass-scaling functions we developed (Table 4) are valuable in that they facilitate calculation of echinoid community biomass from field surveys, given surveys include data on echinoid identities, densities, and test diameters. Biomass (vs. density) of fishes is widely-accepted by scientists and managers as an important ecological metric that drives ecological dynamics in coral reefs [22, 62]; however, the biomass of echinoid communities is rarely assessed, though can be equally important [63]. The lack of echinoid community structure and biomass data in many long-term surveys on coral reefs my impede science and management because, as for fishes, density alone fails to account for variation in community structure and biomass, both of which exert significant influence on energetic demands [1] that can drive consumption rates by herbivore communities.

### Echinoid metabolism

The metabolic rates we measured were similar to those measured in previous studies in different regions using similar species and methods. For example, Moulin et al. 2015 reported mean metabolic rates for *E*. *matthaei* on Reunion Island of 0.016 mgO_2_/g/h [64], nearly identical to our 0.015 mgO_2_/g/h for the same species in Hawaii. Idrisi et al. 2016 reported mean metabolic rates for *Diadema antellarum* (a diadematid) in the Florida Keys as 0.035 mgO_2_/g/h, similar to the 0.034 mgO_2_/g/h of *E*. *calamaris* (also a diadematid) that we measured in Hawaii. Interestingly, these echinoid metabolic rates are all approximately 10-fold lower than values reported for a common herbivorous parrotfish, *Sparisoma viridens*, in the Caribbean (0.2 mgO_2_/g/h) [65]. The similarity of our results to previous measurements of echinoid metabolism is remarkable given they were all measured in different ocean basins (Pacific, Indian, and Caribbean) using different methodologies. This suggests that the metabolic rates and scaling parameters we report here are reasonable estimates for the metabolic scaling of tropical herbivorous echinoids on coral reefs around the world.

The scaling exponent for individual metabolic rate (*γ* = 0.44) did not differ significantly among urchin species, however did differ significantly from the 3/4 (*γ* = 0.75) ‘universal’ scaling exponent of the MTE. Therefore, individual metabolic rates of echinoids increased much slower (as a function of mass) than predicted by general metabolic theory [2]. Similarly, the scaling exponent for biomass-specific metabolic rate (*α* = -0.56) was significantly lower than the -1/4 (*α* = -0.25) universal scaling exponent of the MTE. Therefore, mass-specific metabolism decreased significantly faster with mass than MTE would predict. However, some researchers have suggested that a scaling exponent of 2/3 (0.67) maybe be appropriate for many taxa [66] and, though our estimates are much lower than 0.67, they do not deviate significantly from this prediction. The low scaling exponents we report here are supported by previous values (e.g., *γ* = 0.61) observed for echinoderms [57], thus indicating that echinoid metabolism declines rapidly with size and is lower for large echinoids than predicted by classic theory. This is likely due to the large contribution of metabolically-inactive calcified skeleton or reduced metabolic activity of soft tissues as echinoids age and grow. Further physiological studies are needed to identify the mechanisms that determine the low metabolic scaling exponent for echinoids.

Skeletal morphology differed greatly among echinoid species and likely contributed to differences in metabolic scaling. For this reason, some researchers have used ash free dry mass (AFDM) to control for skeletal mass, though this is not regularly done across all taxonomic groups [67]. In contrast with our results, when AFDM has been used on echinoids [68] and other echinoderms [69], results appeared to support ¾ scaling. Thus removal of skeletal influence may facilitate a better match between echinoderms and theoretical scaling exponents; however, the need for this additional treatment highlights their unique metabolic ecology as whole, living organisms. Because our organisms were released alive, we could not measure AFDM; however we did examine echinoid metabolism as a function of test volume, which served as a proxy for the quantity of metabolically-active visceral tissue. Scaling exponents based on mass and volume were similar for individual metabolic rates, and relative magnitudes of intercepts were also similar, suggesting that the exoskeleton, alone, did not drive large differences in scaling parameters (Table 3, S4 Fig). However, HM (with its robust calcified spines) exhibited a significantly higher intercept than EM as a function of volume, indicating that HM exhibits a higher metabolic rate in soft tissues than EM, a pattern that was masked by differences in skeletal morphology. While the effects of skeletal material on scaling parameters is interesting, standard models in MTE typically do not correct for variation in skeletal features among most taxa [2], and the ecological and metabolic costs associated with generating and transporting robust skeletons are included as important aspects of an organism’s metabolic ecology [67].

### Echinoid metabolism and metabolic theory

Though significantly different from Kleiber’s 0.75 and -0.25 (for γ and α, respectively), the echinoid scaling coefficients we measured did not differ significantly from predictions of 2/3 scaling [66]. Though nearly a century of research, several seminal books, and modern syntheses all support a ‘universal’ mass scaling exponent (γ) of ¾ for individual metabolic rate versus mass; this value represents an average of exponents that vary in nature. Several studies (including ours) have measured scaling exponents that differ significantly from Kleiber’s Law [53, 70]. For example, other calcified marine invertebrates (e.g. echinoderms and bryozoans) exhibit low mass-scaling exponents (γ = 0.61 and 0.47, respectively) [57, 71], similar to the value we report here for tropical echinoids (γ = 0.44). Allometric scaling of metabolic rates for mammals can also be significantly lower than Kleiber’s Law [72] and vary as a function of the mean mass and taxonomy (e.g., order) of the organism [73]. Furthermore, unicellular heterotrophs differ from Kleiber’s Law in the opposite direction from small mammals, with linear (γ = 1) scaling for protists and even superlinear (γ = 2) scaling for bacteria [74]. Though results from previous studies of metabolic scaling for echinoderms could support γ values of either 2/3 or 3/4 [70], our results provide support for the former, or perhaps even lower values.

The routine metabolic rates we present in this study did not include a starvation pretreatment; therefore, it is possible that natural variability in postprandial metabolism influenced our results [50], as would be observed in measures of field metabolic rates. Interestingly, studies comparing field versus resting metabolic rates of terrestrial ectotherms suggest that field conditions greatly increase scaling exponents [52], which could not explain the low values we observed. Furthermore, the metabolic rates we provide are supported by known differences in feeding and behavior and closely match values from previous studies on similar species in different habitats. Our statistical results also indicate much larger inter- versus intra-specific variation in metabolic rate, suggesting limited effects of individual variation in behavior or recent feeding history. Thus the metabolic rates (and scaling coefficients) we report here appear to be well-supported characterizations of these (and similar) tropical herbivorous echinoids.

Variation among studies in observed metabolic scaling exponents could arise due to differences in intra- vs. interspecific scaling relationships [75] as well as real variation among taxonomic groups, size- and age-classes, and habitats [76]. The pooled scaling exponent we report here reflects the mean intraspecific value for all five species assayed. In contrast, using species means of mass and metabolic rate, we observed a mass-scaling exponent of 0.875; however power was limited by the number species (N = 5) and this result was not significant or informative. To further address these considerations, future comparative studies on the mass-scaling of tropical echinoids would benefit from (a) large samples sizes that maximize the number of species, size ranges, and sample sizes of each species, (b) multiple comparisons of starved vs. unstarved individuals, intra- vs. inter-specific metabolic scaling, and basal vs. field metabolic rates, and (c) comparison of the metabolic responses of different species to environmental changes such as warming, oxygen depletion, and acidification. Due to many logistical tradeoffs, it is unlikely, however, that any one study could achieve all of these goals; therefore many well-controlled and comparable studies are likely needed.

## Conclusion

Echinoderms exert strong top-down effects on benthic dynamics in a variety different marine ecosystems. Predation by asteroids and herbivory by echinoids are often dominant structuring forces in benthic marine ecosystems, both intertidal and subtidal, and in tropical temperate, and polar seas [3–7]. Due to the strong interaction strengths imposed by this diverse group of organisms, it is important that we examine variation in the ecological functions of coexisting taxa to improve our understanding and management of ecosystems [8]. Here, we have provided new information on species-level variation in the scaling of mass and metabolism for 5 herbivorous echinoids common to coral reefs around the globe. Echinoid species exhibited large differences in individual and mass-specific metabolic rates, and this variation in metabolism corresponded with observed differences in behavior and ecology. Such variation in metabolic ecology suggests that these echinoid species exhibit distinct ecological functions.

Metabolic theories have contributed greatly to our understanding of how the biomass and metabolism of species and communities influence ecosystem dynamics [2]. In coral reefs, for example, metabolic rates have been used to estimate the *in situ* contributions of cryptic organisms (i.e., cryptofauna) to total grazing budgets, yielding large consumption estimates (>30% of the daily production) for this under-studied herbivore community [77]. Furthermore, changes in metabolic rates (e.g. due to ocean warming) may result in significant, predictable changes to interaction strengths and ecosystem dynamics [46, 49, 78]. Given the importance of echinoids in coral reefs and the many ecological rates that correlate strongly with biomass and metabolism, data on taxon-specific biomass and metabolism in coral reef studies remains an important gap in our understanding of echinoid community structure and function. The parameters we have provided here (Table 4, Fig 4), combined with survey data on echinoid sizes, community structure, and density, will improve estimates of *in situ* ecological rates, interactions strengths, and will allow researchers to test and model ecological dynamics in new ways and at larger and more ecologically-relevant scales.

## Supporting information

S1 Table Echinoid assay data. (DOCX)

S1 Fig Mean sizes of echinoids used in metabolic assays. (DOCX)

S2 Fig Mean microbial versus uncorrected echinoid oxygen consumption rates. (DOCX)

S3 Fig Comparisons of replicate metabolic assays. (DOCX)

S4 Fig Comparison of mass and volumetric scaling of metabolism. (DOCX)
