# Supplementary material for: Comparative metabolic ecology of tropical herbivorous echinoids on a coral reef
Source: PLoS One. 2018 Jan 18;13(1):e0190470. doi: 10.1371/journal.pone.0190470 (PMC5773235; doi:10.1371/journal.pone.0190470)
Supplement: S1 Table — (DOCX) [file pone.0190470.s001.docx]

S1 Table. Echinoid Assay Data

Table S1.1 Definitions of echinoid metabolic assay data columns

| **Column Title** | **Description** |
| --- | --- |
| Date | date |
| Assay | Urchin species: *E. calamaris*-EC, *E. diadema*-ED, *E. matthaei*-EM, *H. mammillatus*-HM, *T. gratilla*-TG. |
| Ti | initial time |
| Tf | final time |
| Treat | treatment: control-CN , urchin-UR |
| Rep | replicate |
| Chamber | chamber number |
| Temp | temperature (°C) |
| O2i | initial oxygen concentration (mg/l) |
| O2f | final oxygen concentration (mg/l) |
| UT | urchin test diameter (cm) |
| UV | urchin volume (ml) |
| UM | urchin mass (g) |
| V | chamber volume (l) |
| dT | change in time (h) |
| -dO2 | change in oxygen concentration (mg/l) |
| -dO2c | corrected change in oxygen concentration (mg/l) |
| MRc | metabolic rate (mgO2/h) |
| MSMRc | mass-specific metabolic rate (mgO2/g/h) |

Table S1.2 Results of all echinoid metabolic assays

| **Date** | **Assay** | **Ti** | **Tf** | **Treat** | **Rep** | **Chamber** | **Temp** | **O2i** | **O2f** | **UT** | **UV** | **UM** | **V** | **dT** | **-dO2** | **-dO2c** | **MRc** | **MSMRc** |
| --- | --- | --- | --- | --- | --- | --- | --- | --- | --- | --- | --- | --- | --- | --- | --- | --- | --- | --- |
| 7/11/2014 | EC | 11:38 | 12:46 | CN | 1 | 1 | 26.3 | 6.760 | 6.730 | NA | NA | NA | 11.700 | 1.133 | 0.351 | NA | NA | NA |
| 7/11/2014 | EC | 11:38 | 12:46 | CN | 1 | 2 | 26.3 | 6.760 | 6.750 | NA | NA | NA | 11.700 | 1.133 | 0.117 | NA | NA | NA |
| 7/11/2014 | EC | 11:38 | 12:46 | CN | 1 | 3 | 26.3 | 6.770 | 6.750 | NA | NA | NA | 11.700 | 1.133 | 0.234 | NA | NA | NA |
| 7/11/2014 | EC | 12:56 | 13:58 | UR | 1 | 1 | 26.3 | 6.740 | 6.400 | 6.000 | 113.040 | 100.000 | 11.700 | 1.033 | 3.978 | 3.666 | 3.548 | 0.035 |
| 7/11/2014 | EC | 12:56 | 13:58 | UR | 1 | 2 | 26.3 | 6.740 | 6.300 | 6.300 | 130.858 | 127.000 | 11.700 | 1.033 | 5.148 | 4.836 | 4.680 | 0.037 |
| 7/11/2014 | EC | 12:56 | 13:58 | UR | 1 | 3 | 26.3 | 6.730 | 6.400 | 6.000 | 113.040 | 103.000 | 11.700 | 1.033 | 3.861 | 3.549 | 3.435 | 0.033 |
| 7/11/2014 | EC | 14:12 | 15:21 | UR | 2 | 1 | 26.3 | 6.730 | 6.460 | 5.600 | 91.906 | 78.000 | 11.700 | 1.150 | 3.159 | 2.847 | 2.476 | 0.032 |
| 7/11/2014 | EC | 14:12 | 15:21 | UR | 2 | 2 | 26.3 | 6.740 | 6.340 | 5.900 | 107.482 | 90.000 | 11.700 | 1.150 | 4.680 | 4.368 | 3.798 | 0.042 |
| 7/11/2014 | EC | 14:12 | 15:21 | UR | 2 | 3 | 26.3 | 6.720 | 6.350 | 6.300 | 130.858 | 114.000 | 11.700 | 1.150 | 4.329 | 4.017 | 3.493 | 0.031 |
| 7/11/2014 | EC | 15:37 | 16:43 | UR | 3 | 1 | 26.3 | 6.720 | 6.320 | 6.100 | 118.787 | 115.000 | 11.700 | 1.100 | 4.680 | 4.368 | 3.971 | 0.035 |
| 7/11/2014 | EC | 15:37 | 16:43 | UR | 3 | 2 | 26.3 | 6.720 | 6.330 | 6.500 | 143.720 | 120.000 | 11.700 | 1.100 | 4.563 | 4.251 | 3.865 | 0.032 |
| 7/11/2014 | EC | 15:37 | 16:43 | UR | 3 | 3 | 26.3 | 6.700 | 6.320 | 6.200 | 124.725 | 118.000 | 11.700 | 1.100 | 4.446 | 4.134 | 3.758 | 0.032 |
| 7/11/2014 | EC | 16:57 | 18:03 | CN | 2 | 1 | 26.3 | 6.700 | 6.660 | NA | NA | NA | 11.700 | 1.100 | 0.468 | NA | NA | NA |
| 7/11/2014 | EC | 16:57 | 18:03 | CN | 2 | 2 | 26.3 | 6.700 | 6.660 | NA | NA | NA | 11.700 | 1.100 | 0.468 | NA | NA | NA |
| 7/11/2014 | EC | 16:57 | 18:03 | CN | 2 | 3 | 26.3 | 6.690 | 6.670 | NA | NA | NA | 11.700 | 1.100 | 0.234 | NA | NA | NA |
| 6/9/2014 | TG | 13:09 | 14:17 | CN | 1 | 1 | 26.3 | 6.780 | 6.750 | NA | NA | NA | 11.700 | 1.133 | 0.351 | NA | NA | NA |
| 6/9/2014 | TG | 13:09 | 14:17 | CN | 1 | 2 | 26.3 | 6.770 | 6.750 | NA | NA | NA | 11.700 | 1.133 | 0.234 | NA | NA | NA |
| 6/9/2014 | TG | 13:09 | 14:17 | CN | 1 | 3 | 26.3 | 6.760 | 6.750 | NA | NA | NA | 11.700 | 1.133 | 0.117 | NA | NA | NA |
| 6/9/2014 | TG | 14:28 | 15:36 | UR | 1 | 1 | 26.3 | 6.740 | 6.400 | 6.800 | 164.553 | 135.000 | 11.700 | 1.133 | 3.978 | 3.666 | 3.235 | 0.024 |
| 6/9/2014 | TG | 14:28 | 15:36 | UR | 1 | 2 | 26.3 | 6.730 | 6.470 | 6.800 | 164.553 | 142.000 | 11.700 | 1.133 | 3.042 | 2.730 | 2.409 | 0.017 |
| 6/9/2014 | TG | 14:28 | 15:36 | UR | 1 | 3 | 26.3 | 6.730 | 6.440 | 7.200 | 195.333 | 158.000 | 11.700 | 1.133 | 3.393 | 3.081 | 2.719 | 0.017 |
| 6/9/2014 | TG | 15:48 | 16:55 | UR | 2 | 1 | 26.3 | 6.720 | 6.300 | 7.300 | 203.586 | 181.000 | 11.700 | 1.117 | 4.914 | 4.602 | 4.121 | 0.023 |
| 6/9/2014 | TG | 15:48 | 16:55 | UR | 2 | 2 | 26.3 | 6.710 | 6.390 | 7.500 | 220.781 | 191.000 | 11.700 | 1.117 | 3.744 | 3.432 | 3.073 | 0.016 |
| 6/9/2014 | TG | 15:48 | 16:55 | UR | 2 | 3 | 26.3 | 6.710 | 6.360 | 6.800 | 164.553 | 139.000 | 11.700 | 1.117 | 4.095 | 3.783 | 3.388 | 0.024 |
| 6/9/2014 | TG | 17:07 | 18:14 | UR | 3 | 1 | 26.3 | 6.680 | 6.280 | 7.400 | 212.067 | 180.000 | 11.700 | 1.117 | 4.680 | 4.368 | 3.912 | 0.022 |
| 6/9/2014 | TG | 17:07 | 18:14 | UR | 3 | 2 | 26.3 | 6.680 | 6.390 | 6.500 | 143.720 | 117.000 | 11.700 | 1.117 | 3.393 | 3.081 | 2.759 | 0.024 |
| 6/9/2014 | TG | 17:07 | 18:14 | UR | 3 | 3 | 26.3 | 6.680 | 6.370 | 7.100 | 187.307 | 146.000 | 11.700 | 1.117 | 3.627 | 3.315 | 2.969 | 0.020 |
| 6/9/2014 | TG | 18:32 | 19:38 | CN | 2 | 1 | 26.3 | 6.700 | 6.650 | NA | NA | NA | 11.700 | 1.100 | 0.585 | NA | NA | NA |
| 6/9/2014 | TG | 18:32 | 19:38 | CN | 2 | 2 | 26.3 | 6.680 | 6.650 | NA | NA | NA | 11.700 | 1.100 | 0.351 | NA | NA | NA |
| 6/9/2014 | TG | 18:32 | 19:38 | CN | 2 | 3 | 26.3 | 6.680 | 6.660 | NA | NA | NA | 11.700 | 1.100 | 0.234 | NA | NA | NA |
| 6/8/2014 | EM | 13:06 | 14:16 | CN | 1 | 1 | 26.2 | 6.800 | 6.750 | NA | NA | NA | 11.700 | 1.167 | 0.585 | NA | NA | NA |
| 6/8/2014 | EM | 13:06 | 14:16 | CN | 1 | 2 | 26.2 | 6.790 | 6.760 | NA | NA | NA | 11.700 | 1.167 | 0.351 | NA | NA | NA |
| 6/8/2014 | EM | 13:06 | 14:16 | CN | 1 | 3 | 26.2 | 6.790 | 6.750 | NA | NA | NA | 11.700 | 1.167 | 0.468 | NA | NA | NA |
| 6/8/2014 | EM | 15:48 | 16:54 | UR | 1 | 1 | 26.2 | 6.770 | 6.660 | 4.200 | 38.773 | 33.090 | 11.700 | 1.100 | 1.287 | 0.760 | 0.691 | 0.021 |
| 6/8/2014 | EM | 15:48 | 16:54 | UR | 1 | 2 | 26.2 | 6.750 | 6.660 | 4.100 | 36.069 | 38.110 | 11.700 | 1.100 | 1.053 | 0.526 | 0.479 | 0.013 |
| 6/8/2014 | EM | 15:48 | 16:54 | UR | 1 | 3 | 26.2 | 6.740 | 6.670 | 4.100 | 36.069 | 35.166 | 11.700 | 1.100 | 0.819 | 0.293 | 0.266 | 0.008 |
| 6/8/2014 | EM | 17:05 | 18:12 | UR | 2 | 1 | 26.2 | 6.730 | 6.630 | 4.200 | 38.773 | 33.900 | 11.700 | 1.117 | 1.170 | 0.644 | 0.576 | 0.017 |
| 6/8/2014 | EM | 17:05 | 18:12 | UR | 2 | 2 | 26.2 | 6.730 | 6.660 | 3.800 | 28.716 | 25.840 | 11.700 | 1.117 | 0.819 | 0.293 | 0.262 | 0.010 |
| 6/8/2014 | EM | 17:05 | 18:12 | UR | 2 | 3 | 26.2 | 6.720 | 6.650 | 4.300 | 41.609 | 38.190 | 11.700 | 1.117 | 0.819 | 0.292 | 0.262 | 0.007 |
| 6/8/2014 | EM | 14:27 | 15:37 | UR | 3 | 1 | 26.2 | 6.780 | 6.690 | 3.900 | 31.044 | 28.250 | 11.700 | 1.167 | 1.053 | 0.526 | 0.451 | 0.016 |
| 6/8/2014 | EM | 14:27 | 15:37 | UR | 3 | 2 | 26.2 | 6.780 | 6.690 | 4.000 | 33.493 | 26.390 | 11.700 | 1.167 | 1.053 | 0.526 | 0.451 | 0.017 |
| 6/8/2014 | EM | 14:27 | 15:37 | UR | 3 | 3 | 26.2 | 6.780 | 6.660 | 4.100 | 36.069 | 30.310 | 11.700 | 1.167 | 1.404 | 0.878 | 0.752 | 0.025 |
| 6/8/2014 | EM | 18:24 | 19:31 | CN | 2 | 1 | 26.2 | 6.730 | 6.670 | NA | NA | NA | 11.700 | 1.117 | 0.702 | NA | NA | NA |
| 6/8/2014 | EM | 18:24 | 19:31 | CN | 2 | 2 | 26.2 | 6.720 | 6.680 | NA | NA | NA | 11.700 | 1.117 | 0.468 | NA | NA | NA |
| 6/8/2014 | EM | 18:24 | 19:31 | CN | 2 | 3 | 26.2 | 6.720 | 6.670 | NA | NA | NA | 11.700 | 1.117 | 0.585 | NA | NA | NA |
| 6/7/2014 | HM | 11:38 | 12:50 | CN | 1 | 1 | 26.2 | 6.810 | 6.800 | NA | NA | NA | 11.700 | 1.200 | 0.117 | NA | NA | NA |
| 6/7/2014 | HM | 11:38 | 12:50 | CN | 1 | 2 | 26.2 | 6.810 | 6.800 | NA | NA | NA | 11.700 | 1.200 | 0.117 | NA | NA | NA |
| 6/7/2014 | HM | 11:38 | 12:50 | CN | 1 | 3 | 26.2 | 6.810 | 6.800 | NA | NA | NA | 11.700 | 1.200 | 0.117 | NA | NA | NA |
| 6/7/2014 | HM | 13:02 | 14:09 | UR | 1 | 1 | 26.2 | 6.800 | 6.670 | 5.000 | 65.417 | 146.000 | 11.700 | 1.117 | 1.521 | 1.268 | 1.135 | 0.008 |
| 6/7/2014 | HM | 13:02 | 14:09 | UR | 1 | 2 | 26.2 | 6.800 | 6.660 | 5.400 | 82.406 | 173.000 | 11.700 | 1.117 | 1.638 | 1.385 | 1.240 | 0.007 |
| 6/7/2014 | HM | 13:02 | 14:09 | UR | 1 | 3 | 26.2 | 6.790 | 6.650 | 5.900 | 107.482 | 200.000 | 11.700 | 1.117 | 1.638 | 1.385 | 1.240 | 0.006 |
| 6/7/2014 | HM | 14:24 | 15:30 | UR | 2 | 1 | 26.2 | 6.780 | 6.650 | 5.300 | 77.912 | 180.000 | 11.700 | 1.100 | 1.521 | 1.268 | 1.152 | 0.006 |
| 6/7/2014 | HM | 14:24 | 15:30 | UR | 2 | 2 | 26.2 | 6.770 | 6.660 | 4.800 | 57.876 | 129.000 | 11.700 | 1.100 | 1.287 | 1.034 | 0.940 | 0.007 |
| 6/7/2014 | HM | 14:24 | 15:30 | UR | 2 | 3 | 26.2 | 6.770 | 6.660 | 5.200 | 73.585 | 179.000 | 11.700 | 1.100 | 1.287 | 1.034 | 0.940 | 0.005 |
| 6/7/2014 | HM | 15:42 | 16:50 | UR | 3 | 1 | 26.2 | 6.770 | 6.670 | 5.100 | 69.421 | 175.000 | 11.700 | 1.133 | 1.170 | 0.916 | 0.809 | 0.005 |
| 6/7/2014 | HM | 15:42 | 16:50 | UR | 3 | 2 | 26.2 | 6.760 | 6.660 | 5.000 | 65.417 | 115.000 | 11.700 | 1.133 | 1.170 | 0.916 | 0.809 | 0.007 |
| 6/7/2014 | HM | 15:42 | 16:50 | UR | 3 | 3 | 26.2 | 6.760 | 6.670 | 4.700 | 54.334 | 116.000 | 11.700 | 1.133 | 1.053 | 0.800 | 0.705 | 0.006 |
| 6/7/2014 | HM | 17:02 | 18:05 | CN | 2 | 1 | 26.2 | 6.760 | 6.720 | NA | NA | NA | 11.700 | 1.050 | 0.468 | -0.059 | -0.056 | NA |
| 6/7/2014 | HM | 17:02 | 18:05 | CN | 2 | 2 | 26.2 | 6.760 | 6.730 | NA | NA | NA | 11.700 | 1.050 | 0.351 | -0.176 | -0.167 | NA |
| 6/7/2014 | HM | 17:02 | 18:05 | CN | 2 | 3 | 26.2 | 6.750 | 6.720 | NA | NA | NA | 11.700 | 1.050 | 0.351 | NA | NA | NA |
| 6/6/2014 | ED | 12:32 | 13:33 | CN | 1 | 1 | 26.1 | 6.780 | 6.770 | NA | NA | NA | 11.700 | 1.017 | 0.117 | NA | NA | NA |
| 6/6/2014 | ED | 12:32 | 13:33 | CN | 1 | 2 | 26.1 | 6.770 | 6.770 | NA | NA | NA | 11.700 | 1.017 | 0.000 | NA | NA | NA |
| 6/6/2014 | ED | 12:32 | 13:33 | CN | 1 | 3 | 26.1 | 6.770 | 6.780 | NA | NA | NA | 11.700 | 1.017 | -0.117 | NA | NA | NA |
| 6/6/2014 | ED | 14:01 | 15:15 | UR | 1 | 1 | 26.1 | 6.810 | 6.590 | 5.900 | 107.482 | 126.000 | 11.700 | 1.233 | 2.574 | 2.457 | 1.992 | 0.016 |
| 6/6/2014 | ED | 14:01 | 15:15 | UR | 1 | 2 | 26.1 | 6.810 | 6.580 | 5.500 | 87.070 | 81.000 | 11.700 | 1.233 | 2.691 | 2.574 | 2.087 | 0.026 |
| 6/6/2014 | ED | 14:01 | 15:15 | UR | 1 | 3 | 26.1 | 6.810 | 6.670 | 3.800 | 28.716 | 30.000 | 11.700 | 1.233 | 1.638 | 1.521 | 1.233 | 0.041 |
| 6/6/2014 | ED | 15:30 | 16:36 | UR | 2 | 1 | 26.1 | 6.780 | 6.630 | 4.500 | 47.689 | 47.000 | 11.700 | 1.100 | 1.755 | 1.638 | 1.489 | 0.032 |
| 6/6/2014 | ED | 15:30 | 16:36 | UR | 2 | 2 | 26.1 | 6.780 | 6.660 | 3.900 | 31.044 | 31.000 | 11.700 | 1.100 | 1.404 | 1.287 | 1.170 | 0.038 |
| 6/6/2014 | ED | 15:30 | 16:36 | UR | 2 | 3 | 26.1 | 6.770 | 6.590 | 5.900 | 107.482 | 89.000 | 11.700 | 1.100 | 2.106 | 1.989 | 1.808 | 0.020 |
| 6/6/2014 | ED | 16:48 | 17:49 | UR | 3 | 1 | 26.1 | 6.760 | 6.600 | 5.400 | 82.406 | 81.000 | 11.700 | 1.017 | 1.872 | 1.755 | 1.726 | 0.021 |
| 6/6/2014 | ED | 16:48 | 17:49 | UR | 3 | 2 | 26.1 | 6.760 | 6.570 | 5.100 | 69.421 | 79.000 | 11.700 | 1.017 | 2.223 | 2.106 | 2.071 | 0.026 |
| 6/6/2014 | ED | 16:48 | 17:49 | UR | 3 | 3 | 26.1 | 6.750 | 6.620 | 4.900 | 61.570 | 73.000 | 11.700 | 1.017 | 1.521 | 1.404 | 1.381 | 0.019 |
| 6/6/2014 | ED | 18:01 | 19:06 | CN | 2 | 1 | 26.1 | 6.750 | 6.730 | NA | NA | NA | 11.700 | 1.083 | 0.234 | -0.293 | -0.270 | NA |
| 6/6/2014 | ED | 18:01 | 19:06 | CN | 2 | 2 | 26.1 | 6.750 | 6.730 | NA | NA | NA | 11.700 | 1.083 | 0.234 | -0.293 | -0.270 | NA |
| 6/6/2014 | ED | 18:01 | 19:06 | CN | 2 | 3 | 26.1 | 6.750 | 6.730 | NA | NA | NA | 11.700 | 1.083 | 0.234 | NA | NA | NA |
