# Supplementary material for: Comparative metabolic ecology of tropical herbivorous echinoids on a coral reef
Source: PLoS One. 2018 Jan 18;13(1):e0190470. doi: 10.1371/journal.pone.0190470 (PMC5773235; doi:10.1371/journal.pone.0190470)

S1 Fig. Mean sizes of echinoids used in metabolic assays. Echinoid test diameter (A), wet mass (B), and estimated test volume (C). Volume was estimated from test diameters (radius) assuming a hemispherical test morphology (V = 4/3*π*r^3^). Urchin codes as in Fig1a.


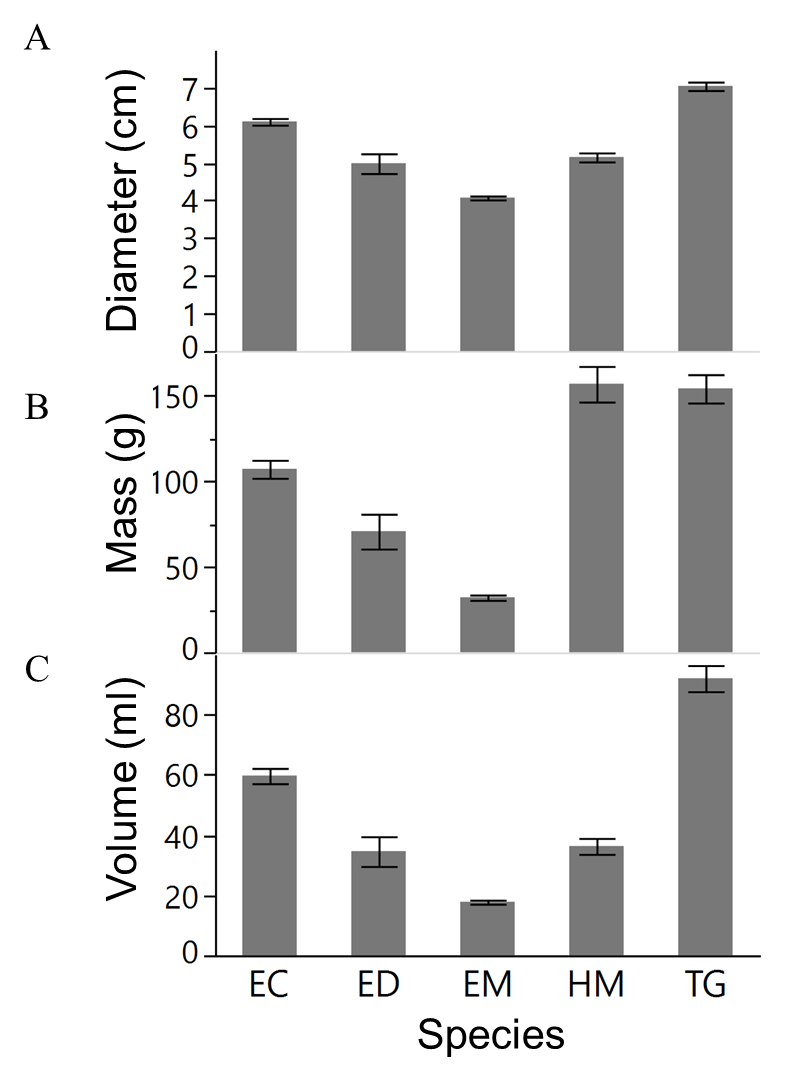

Supplement: S1 Fig — (DOCX) [file pone.0190470.s002.docx]
