# Supplementary material for: Comparative metabolic ecology of tropical herbivorous echinoids on a coral reef
Source: PLoS One. 2018 Jan 18;13(1):e0190470. doi: 10.1371/journal.pone.0190470 (PMC5773235; doi:10.1371/journal.pone.0190470)
Supplement: S2 Fig — (DOCX) [file pone.0190470.s003.docx]

S2 Fig. Mean microbial versus uncorrected echinoid oxygen consumption rates. Background levels of microbial respiration (CN, dashed line) relative to uncorrected values of echinoid assays. All values are the mean ± SE. Urchin codes as in Fig1a. Rates in echinoid assays varied greatly among species (approximately 1-4 mgO_2_/h) and remained well above background levels of microbial respiration (0.27 ± 0.03 mgO_2_/h). Echinoid metabolic rates were corrected to account for background levels of microbial respiration.


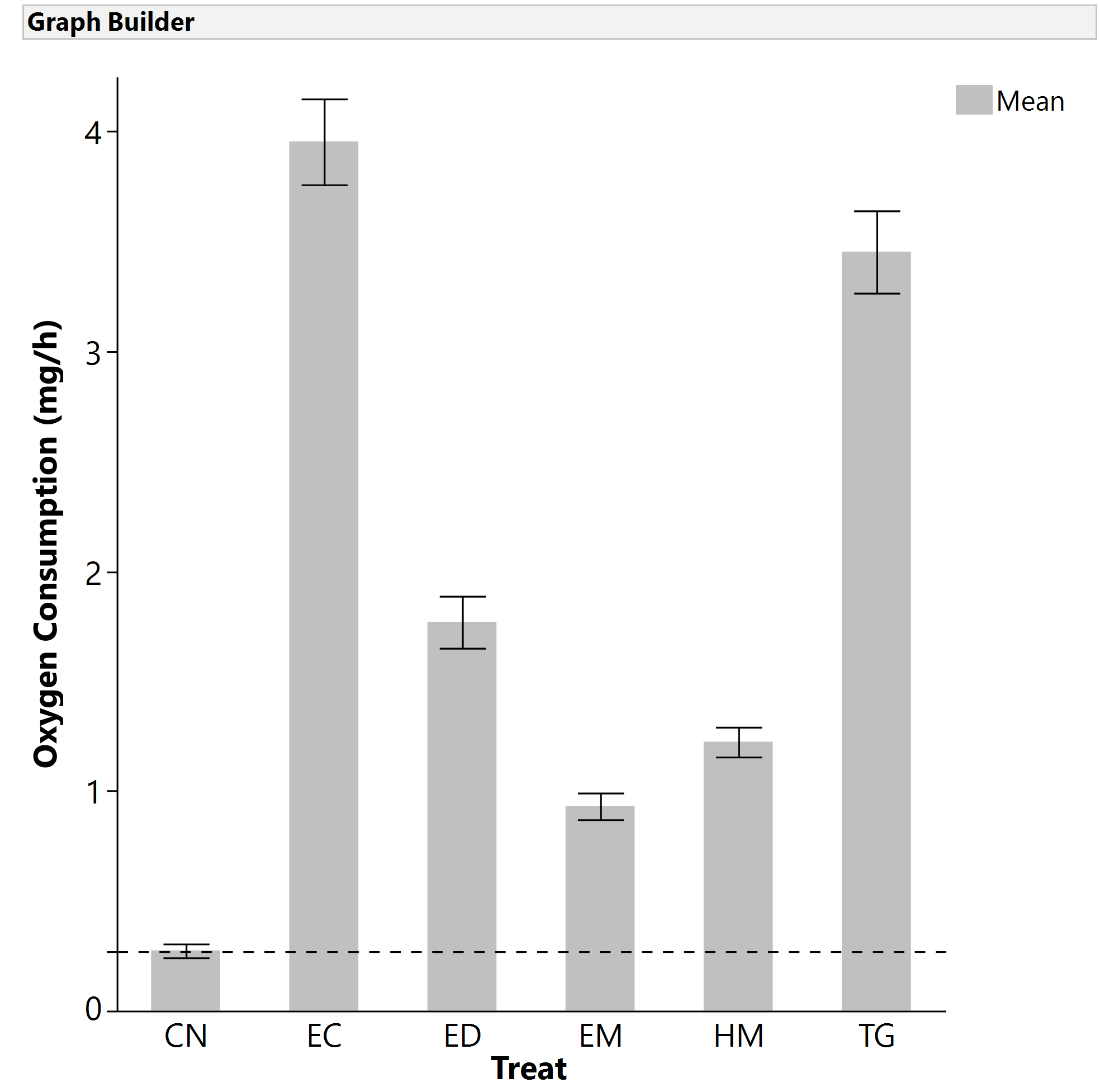


Treatment
