# Supplementary material for: Comparative metabolic ecology of tropical herbivorous echinoids on a coral reef
Source: PLoS One. 2018 Jan 18;13(1):e0190470. doi: 10.1371/journal.pone.0190470 (PMC5773235; doi:10.1371/journal.pone.0190470)
Supplement: S3 Fig — (DOCX) [file pone.0190470.s004.docx]

S3 Fig. Comparisons of replicate metabolic assays. Mean ± SE control-corrected total metabolic rates (TMR) for each of 3 replicate (n=3) assays for EC (A), ED (B), EM (C), HM (D) and TG (E). Urchin codes as in Fig1a. No significant differences among replicate assays were observed for 4/5 echinoid species; with small differences (Δ = 0.19-0.43 mgO2/h) observed between replicate HM assays (F_2,6_ = 19.5, p = 0.002). Given the small difference for HM, and lack of any significant difference for all other echinoids, we concluded that results were not affected by order or time of day.


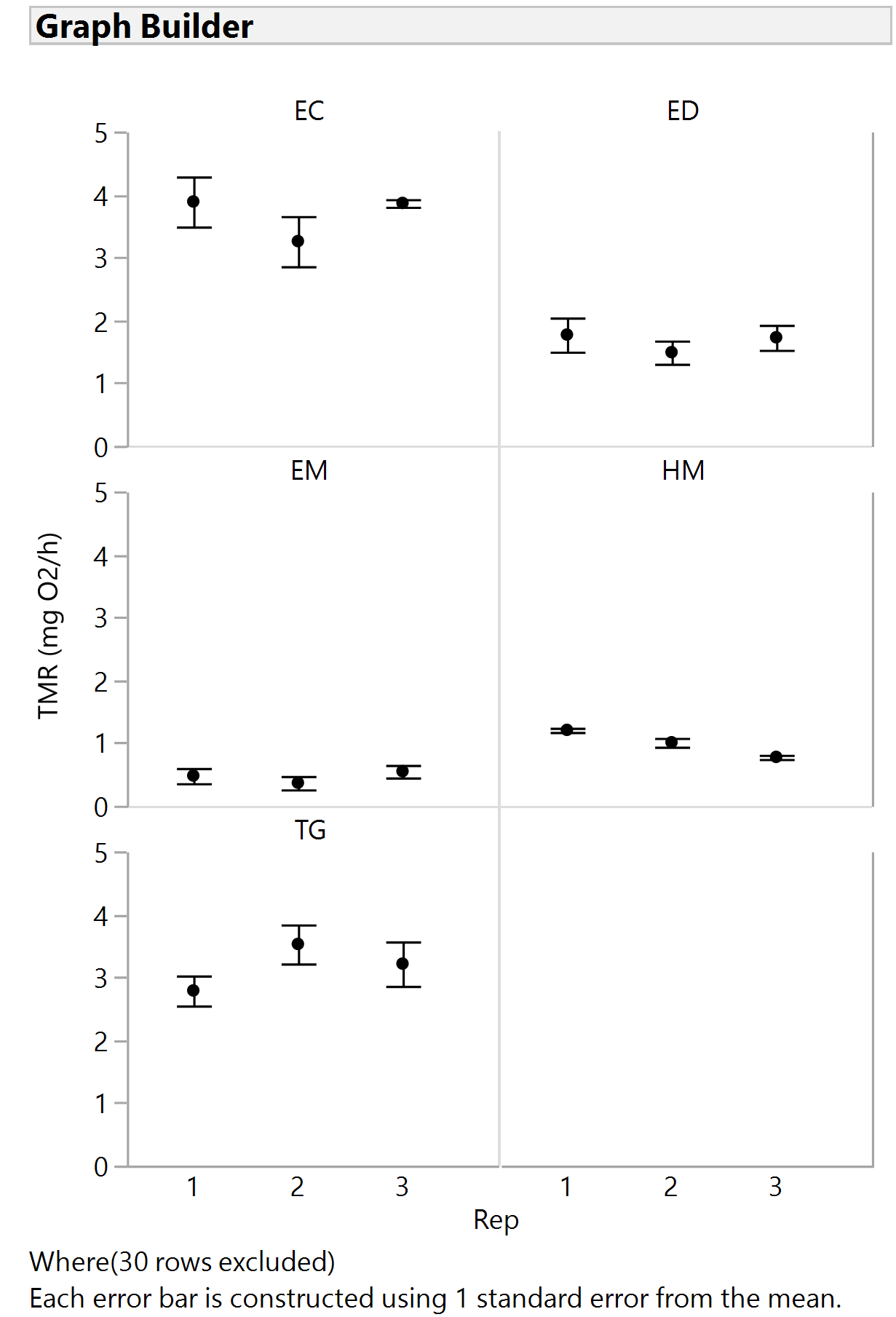


A

D

C

E

B
