# Supplementary material for: Comparative metabolic ecology of tropical herbivorous echinoids on a coral reef
Source: PLoS One. 2018 Jan 18;13(1):e0190470. doi: 10.1371/journal.pone.0190470 (PMC5773235; doi:10.1371/journal.pone.0190470)
Supplement: S4 Fig — (DOCX) [file pone.0190470.s005.docx]

**S4 Fig. Comparison of mass and volumetric scaling of metabolism.** Log-transformed total metabolic rate (*I*, mgO_2_/h) versus (A) echinoid mass (*M*, g) and (B) volume (*V*, ml). Relationships between echinoid mass and volume (C). Urchin codes as in Fig1a. Due to the large differences in mass-diameter scaling relationships, we examined the metabolic scaling of echinoids in relation to test volume (*V*), which served as a proxy for the quantity of visceral mass independent of skeletal mass. Volume was modeled as a hemisphere (*V* = 0.5*4/3*pi*radius^3^). Log(*M*) alone proved a poor overall predictor of individual metabolic rate (A), however log(*V*) alone explained 74% of the variance, without species identity (B). Due to the unique, heavily calcified skeleton and spines of *H. mammillatus*, this species was a unique outlier with respect to mass scaling (C), but fit the overall metabolic model fairly well with respect to *V*. Thus the relatively low mass-scaling observed for *H. mammillatus* was likely driven by its heavy, metabolically-inactive skeleton, and the low overall mass-scaling relationships for all echinoderms was also likely influenced by their robust skeletal morphology.

**
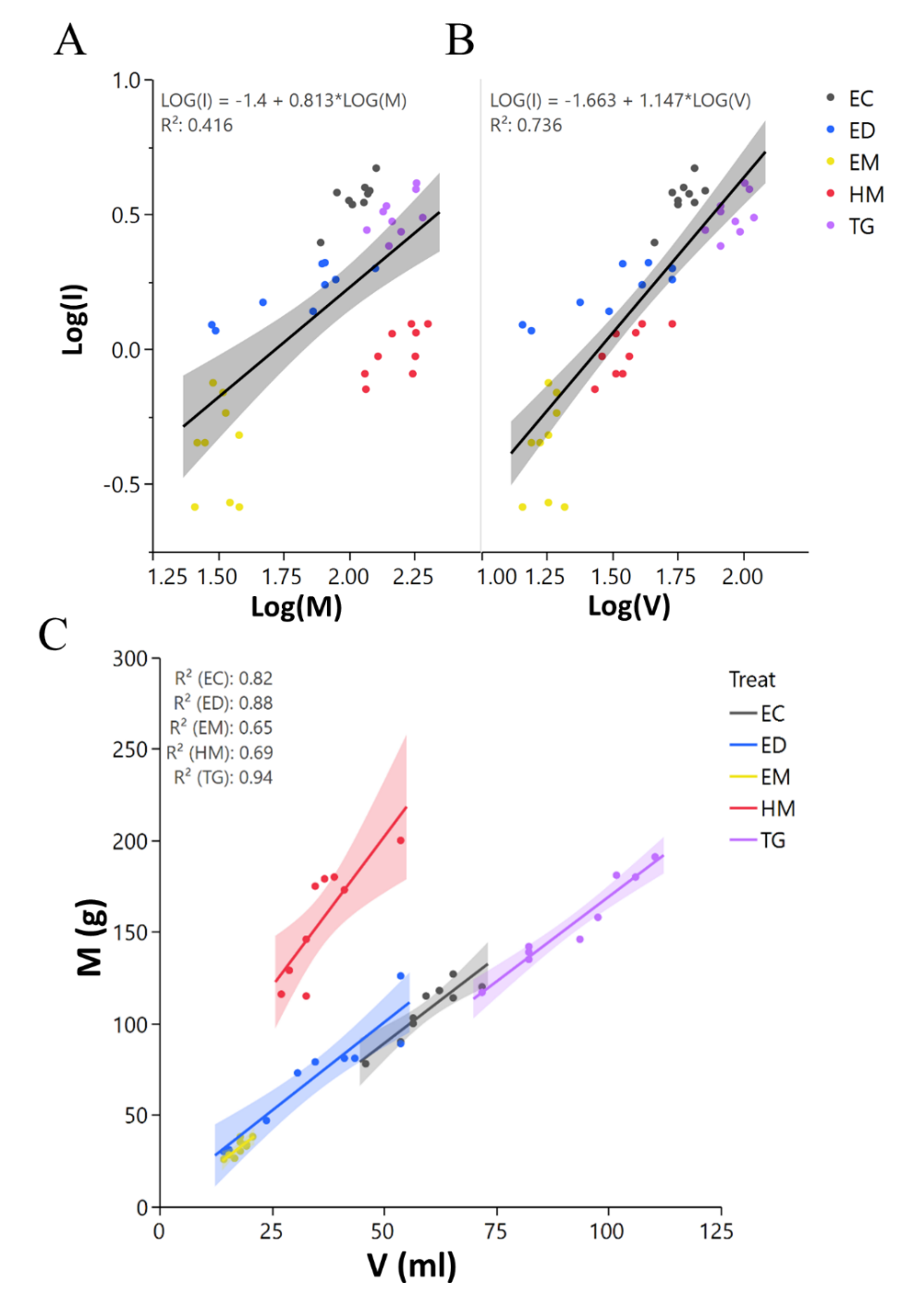
**
